# Seroprevalence of hepatitis A virus infection in urban and rural areas in Vietnam

**DOI:** 10.1371/journal.pone.0323139

**Published:** 2025-05-16

**Authors:** Nguyen Thi Cam Huong, Dang Van Tri, Nguyen Huy Luan, Nguyen Huu Thinh, Tran Song Ngoc Chau, Truc Ly Thi Vo, Dang Khoa Tran, Minh Nguyen, Adriana Guzman-Holst

**Affiliations:** 1 Department of Infectious Diseases, University of Medicine and Pharmacy at Ho Chi Minh City, Ho Chi Minh City, Vietnam; 2 Pediatric Department, University of Medicine and Pharmacy at Ho Chi Minh City, Ho Chi Minh City, Vietnam; 3 Department of Health Screening, University Medical Center at Ho Chi Minh City, Ho Chi Minh City, Vietnam; 4 Faculty of Medicine and Pharmacy, Tay Nguyen University, Buon Ma Thuot, Dak Lak, Vietnam; 5 GSK, Medical Affairs, Ho Chi Minh City, Vietnam; 6 GSK, Real-world Evidence & Health Outcomes Research, Wavre, Belgium; East Carolina University Brody School of Medicine, UNITED STATES OF AMERICA

## Abstract

**Background/objectives:**

The prevalence of hepatitis A virus (HAV) is associated with socioeconomic conditions, access to clean drinking water, and improvements in sanitation. In Vietnam, epidemiological data on HAV have been limited over the past two decades. This study aims to assess age-specific HAV seroprevalence across two distinct geographic regions, urban and rural areas, and identify the risk factors associated with HAV seropositivity in Vietnam.

**Methods:**

This cross-sectional seroprevalence study was conducted in two distinct areas in Vietnam. Serological testing for anti-HAV total antibodies was performed, and socio-demographic questionnaires were administered to all participants. The age at the midpoint of population immunity (AMPI) was calculated and analyzed.

**Results:**

A total of 1,281 participants aged 1–80 years were included, with 649 from urban areas and 632 from rural areas. Of the total participants, 33.2% were aged <15 years. Overall, HAV seropositivity was 69.2%, with urban areas exhibiting significantly lower seropositivity (57.9%) compared to rural areas (80.7%) (p < 0.001). The AMPI was 29 years, indicating Vietnam is at intermediate HAV endemicity. Multivariate analysis identified key risk factors for HAV infection, including age and rural residence. Conversely, participants with higher educational levels and those who consumed boiled drinking water were less likely to be HAV seropositive.

**Conclusions:**

The study identified significant differences in the HAV seroprevalence between urban and rural areas, providing critical data for public health officials. These findings highlight the key role of targeted public health interventions and vaccination programs in mitigating HAV infection rates and reducing the disease burden, particularly among high-risk populations in Vietnam.

## Introduction

Hepatitis A is an infectious disease caused by the hepatitis A virus (HAV), which is primarily transmitted via the fecal-oral route through contaminated food, water, or direct contact with an infectious individual [1]. Clinical manifestations of HAV range from asymptomatic infection to symptomatic hepatitis, which may include fever, malaise, abdominal discomfort, diarrhea, and jaundice. HAV infection is usually asymptomatic in children (~90% of cases) but commonly symptomatic in adults (>70% of cases) [2].

In 2019, approximately 159 million new cases of HAV infection were reported globally, which resulted in 39,000 deaths and 2.3 million disability-adjusted life years. Despite a 4% increase in global incidence, deaths related to HAV infection significantly decreased by 40% between 2010 and 2019, possibly due to improved healthcare access and sanitation measures [2,3].

The global incidence rate of HAV infection is unevenly distributed, primarily driven by socioeconomic factors, access to clean water, and sanitary and hygiene practices. The disease burden was highest in low- and middle-income countries, accounting for 60% of acute cases of hepatitis A and 97% of global deaths. In particular, Southeast Asia has the highest number of estimated hepatitis A cases, contributing to 26% of global cases, 60% of global deaths, and a mortality rate of 12 deaths per million annually [2–4].

Economic development in many countries has led to a global decline in HAV endemicity, consequently decreasing childhood exposure. However, symptomatic and more severe illnesses have been observed in older adults, especially in regions with moderate to high endemicity [5]. Serological surveys, which measure HAV antibodies in blood, provide reliable estimates of endemicity and disease burden. While middle-income regions in Asia, Latin America, Eastern Europe, and the Middle East currently show intermediate or low endemicity, improvements in water sanitation and hygiene practices may paradoxically increase susceptibility to HAV infections and overall disease burden [2,6–8].

Countries in Southeast Asia exhibit the full spectrum of HAV endemicity, influenced by factors such as access to safe drinking water, which likely aided in epidemiological shifts in the region [9–11]. Recent surveys indicate low endemicity in the Philippines and Indonesia [12,13], while Vietnam appears to be transitioning from high to intermediate endemicity levels. However, there is a lack of updated national or regional HAV seroprevalence data over the past two decades in all Southeast Asian countries except for Thailand [5,10], thereby limiting a comprehensive understanding of these shifts.

This gap is notable in countries including Vietnam, which have experienced significant socioeconomic improvements but still report sporadic outbreaks of varying severities. Therefore, effective preventive measures, including vaccination, are critical. In Vietnam, hepatitis A vaccination is recommended under the “Recommended Vaccination Schedule for All Ages” issued by the Vietnam Preventive Medicine Association. The schedule includes a two-dose regimen for children aged 1–5 years, with an interval of 6–18 months between doses. Currently, two types of vaccines are available: an inactivated adsorbed vaccine introduced in 2010 for active immunization against HAV and a combined inactivated hepatitis A and hepatitis B vaccine introduced in 2012 [14]. However, access to hepatitis A vaccines is limited to the private healthcare sector. Moreover, urban-rural disparities in sanitation and vaccination coverage have likely influenced the HAV epidemiological shift; however, specific data on these trends and the associated infection risk factors in Vietnam are lacking. While urban areas globally have seen a decline in hepatitis A infections, rates in rural areas remain high, often influenced by socioeconomic factors such as hygiene, sanitation, and drinking water quality [15]. Effective preventive strategies for HAV infection require understanding each region’s current endemicity levels and risk factors.

This study aimed to assess age-specific HAV seroprevalence in Vietnam and identify associations between sociodemographic parameters and HAV seroprevalence. The primary objective was to evaluate the age-specific prevalence of HAV infection in two geographically distinct regions: urban and rural areas. The secondary objectives were to determine the age at the midpoint of population immunity (AMPI), that is, the age at which 50% of the population has been infected with HAV, and to assess the strength of associations between known risk factors and HAV seropositivity within the study population. These data are essential for decision-makers to develop targeted and comprehensive strategies to reduce the current and future burden of HAV infection, eventually contributing to the goal of eliminating hepatitis A in Southeast Asia by 2030 [16].

## Materials and methods

This cross-sectional, observational study was conducted from 9 January 2023–25 May 2024 to assess age-specific anti-HAV seroprevalence among Vietnamese inhabitants.

The area-specific study staff and community health officers coordinated population-based age-stratified (non-random) sampling. In the rural areas, five provinces were selected, and study staff collaborated with community health officers to recruit participants from specific age groups, including first-year medical students from the University of Medicine and Pharmacy at Ho Chi Minh City and patients attending liver and vaccination clinics at the University Medical Center (UMC). In the urban population, initial participants included medical students and hospital staff from UMC aged ≥18 years and children from kindergartens, with additional recruitment from liver and vaccination clinics at UMC to ensure the desired age range was represented.

The study was conducted according to the Declaration of Helsinki and the International Ethical Guidelines for Health-related Research Involving Humans, Council for International Organizations of Medical Sciences, Geneva, 2016. The study protocol and associated documents were approved by the Biomedical Research Ethics Board of the University of Medicine and Pharmacy at Ho Chi Minh City, Vietnam (1081/UMP-BOARD). All participants provided written consent for this study.

### Study population

Children and adults aged 1–80 who had resided in the selected geographical areas for at least six months were included if they, or their legal representatives, were willing to participate and provided informed consent. Participants were excluded if they had any specific medical condition that could pose health risks during the study, such as contraindications to blood drawing, receiving blood derivatives, being immunosuppressed, or having terminal illnesses or psychological disorders. Only one participant per household was enrolled.

Based on the World Health Organization (WHO) categories for hepatitis A endemicity studies, the study cohort was stratified into 11 pre-defined age groups s: 1–2, 3–4, 5–9, 10–14, 15–19, 20–24, 25–29, 30–34, 35–39, 40–49, and ≥50 years [2].

### Serological testing and data management

Total HAV antibodies were measured from 3 mL of peripheral blood collected by trained staff using EDTA tubes. The samples were labeled, registered by the study field coordinator, refrigerated at 4 °C, and transported on ice to the designated regional laboratory for processing within 36 hours of collection. Blood samples were processed according to local laboratory procedures, and HAV total antibody testing was conducted within 36 hours of arrival at the laboratory. The serum was stored at -20 °C, if needed for further testing, for up to three months.

Data was collected using standardized paper-based questionnaires. Individual results were entered into a centralized, anonymized database using unique participant identification (ID), with duplicate entries to ensure accuracy. Internal checks were implemented to prevent typing errors and ensure data consistency.

### Outcomes

The primary outcome was the HAV immune status of the study participants, determined by measuring total antibodies (IgG + IgM) against HAV in the peripheral blood samples. This testing helps assess individual’s long-term immunity and the appropriateness of vaccination. Blood samples were analyzed using the Electrochemiluminescence Immunoassay II (ECLIA—Roche Elycis-Cobas II), providing semiquantitative results as reactive (COI ≤ 1.0) or non-reactive (COI > 1.0) [17], with the reference threshold corresponding to 20 IU/ml. Age-specific seroprevalence was calculated as the proportion of individuals with reactive HAV antibody results within each age group.

Exposure was derived from participants’ age at enrollment, confirmed using the date of birth documented in their legal IDs. Predictors of past or recent HAV infection were collected through a specially designed interviewer-administered questionnaire overseen by trained interviewers who assisted participants or their legal guardians. The questionnaire was developed based on a previously validated tool for assessing Hepatitis A risk factors, the WHO/UNICEF core questions on drinking water, sanitation, and hygiene for household surveys; the water/sanitation, assets, maternal education, and income (WAMI) index, and a recent systematic review of sporadic HAV infection risk factors [18–20]. The questionnaire comprised 44 questions divided into five sections: (a) sociodemographic information (8 items), (b) knowledge of hepatitis A (7 items), (c) past medical history of hepatitis (10 items), (d) access to water safety (9 items), and (e) hygienic food intake practices (10 items). For participants aged <14 years, responses were obtained from their parents or legal guardians, (S1 Appendix).

### Sample size

The sample size was calculated based on the age-specific seroprevalence observed in Thailand, a country leading the endemicity shift in the region [9]. The sample size is calculated using the formula:


$$n=(Z^{2}xP(1/P))/e^{2}$$

Where:

Z is the value from standard normal distribution corresponding to the desired confidence level. (Z = 1.64 for 90% confidence interval [CI])

P is the expected proper proportion

And e is the desired precision

Precision was set to 5% for prevalence estimates of ≥10% and 10% for higher prevalence estimates, with 90% CIs. Considering Vietnam’s population pyramid and stratified age groups, 640 participants were recruited per geographic area (urban and rural), allowing for a 10% margin for potential losses or unsuitable blood samples (S1 Table). A stopping rule was applied once the target sample size for each age group was reached. With a total sample size of 1144–1280 participants, the study was powered at 80% with 95% CI to assess the strength of association between known risk factors and HAV seropositivity.

### Statistical analysis

Descriptive statistics were used to calculate seroprevalence by age groups. AMPI, defined as the youngest age at which 50% of the population shows serologic evidence of prior HAV infection, was calculated to obtain a more specific measure of endemicity [6]. Endemicity levels were classified as follows: very high (AMPI <5 years), high (AMPI 5–14 years), intermediate (AMPI 15–34 years), and low (AMPI ≥35 years) [8]. Logarithmic, polynomial, and sigmoidal curves were tested to model the age-specific seroprevalence trend, and the R² estimator 1 was used to select the best-fitting curve. Kaplan-Meier was used to determine the AMPI overall and for each region. The chi-square test was used to compare AMPI between urban and rural areas.

Descriptive statistics were used to summarize the participants’ responses to the purpose-made questionnaire. The statistical significance of differences between urban and rural regions was assessed using the chi-square or Mann-Whitney U test.

Bivariate and multivariate analyses were performed to determine statistical significance between risk factors for HAV infection and past (current) exposure to HAV. Socioeconomic status was estimated using the WAMI index [21] based on survey data. The prevalence ratios (PR) were used to measure the strength of the association between HAV infection risk factors and past exposure through univariate (crude) log-binomial models [22]. Factors showing potential association (p < 0.20) were included in the multivariate log-binomial model. Due to the likelihood of collinearity [23], logistic regression with backward elimination was used to determine whether the final model of variables was significantly associated with anti-HAV total reactivity (p < 0.05).

## Results

Of the 1281 study participants, 649 were from urban areas, and 632 were from rural areas. The sociodemographic characteristics differed significantly between urban and rural populations, particularly in education levels, parental education, and occupation. However, age and gender distributions were comparable (Table 1).

**Table 1:** Sociodemographic characteristics in urban and rural participants.

Sociodemographic/ Statistics	Study population (N = 1281)	Urban (n = 649)	Rural (n = 632)	p value^*^
**Gender**				
Male, n (%)	559 (43.6)	280 (43.1)	279 (44.1)	0.781
Female, n (%)	722 (56.4)	369 (55.9)	353 (55.9)	
**Age groups, in years**				
1-2, n (%)	112 (8.7)	57 (8.8)	55 (8.7)	0.959
3-4, n (%)	113 (8.8)	54 (8.3)	59 (9.3)	
5-9, n (%)	101 (7.9)	51 (7.9)	50 (7.9)	
10-14, n (%)	99 (7.7)	52 (8.0)	47 (7.4)	
15-19, n (%)	136 (10.6)	76 (11.7)	60 (9.5)	
20-24, n (%)	153 (11.9)	80 (12.3)	73 (11.6)	
25-29, n (%)	148 (11.6)	76 (11.7)	72 (11.4)	
30-34, n (%)	133 (10.4)	68 (10.5)	65 (10.3)	
35-39, n (%)	126 (9.8)	60 (9.2)	66 (10.4)	
40-49, n (%)	100 (7.8)	48 (7.4)	52 (8.2)	
≥50, n (%)	60 (4.7)	27 (4.2)	33 (5.2)	
**Participants’ education level**				
Primary school, n (%)	166 (13.0)	54 (8.3)	112 (17.7)	<0.001
Middle school, n (%)	162 (12.6)	51 (7.9)	111 (17.6)	
High school, n (%)	169 (13.2)	44 (6.8)	125 (19.8)	
College-University, n (%)	529 (41.3)	378 (58.2)	151 (23.9)	
Illiteracy, n (%)	10 (0.8)	0	10 (1.6)	
<6 year (children), n (%)	245 (19.1)	122 (18.8)	123 (19.5)	
**Occupation**				
Professional, n (%)	77 (6.0)	46 (7.1)	31 (4.9)	<0.001
Semi-professional, n (%)	90 (7.0)	42 (6.5)	48 (7.6)	
Clerical/shop owner, n (%)	56 (4.4)	48 (7.4)	8 (1.3)	
Skilled worker, n (%)	185 (14.4)	117 (18.0)	68 (10.8)	
Semi-skilled worker, n (%)	22 (1.7)	4 (0.6)	18 (2.8)	
Unskilled worker, n (%)	114 (8.9)	14 (2.2)	100 (15.8)	
Household duties, n (%)	70 (5.5)	7 (1.1)	63 (10.0)	
Unemployed (adults), n (%)	11 (0.9)	0	11 (1.7)	
Attending school, n (%)	427 (33.3)	267 (41.1)	160 (25.3)	
Attending garden/pre-school, n (%)	128 (10.0)	56 (8.6)	72 (11.4)	
At home (children), n (%)	101 (7.9)	48 (7.4)	53 (8.4)	
**Father’s education level**				
Primary school, n (%)	276 (21.5)	77 (11.9)	199 (31.5)	<0.001
Middle school, n (%)	279 (21.8)	103 (15.9)	176 (27.8)	
High school, n (%)	336 (26.2)	195 (30.0)	141 (22.3)	
College-University, n (%)	343 (26.8)	264 (40.7)	79 (12.5)	
Illiteracy, n (%)	47 (3.7)	10 (1.5)	37 (5.9)	
**Mother’s education level**				
Primary school, n (%)	302 (23.6)	94 (14.5)	208 (32.9)	<0.001
Middle school, n (%)	297 (23.2)	120 (18.5)	177 (28.0)	
High school, n (%)	302 (23.6)	176 (27.1)	126 (19.9)	
College-University, n (%)	321 (25.1)	249 (38.4)	72 (11.4)	
Illiteracy, n (%)	59 (4.6)	10 (1.5)	49 (7.8)	

n, frequency; ^*^Chi-square test.

Components of the WAMI index differed significantly between urban and rural areas. The urban population showed substantially better assets, maternal education, and income outcomes, while water/sanitation remained comparable between both regions, indicating similar access or quality. The average WAMI was 0.80 ± 0.13 for urban areas and 0.59 ± 0.12 for rural areas (Table 2).

**Table 2:** Differences in WAMI between urban and rural participants.

WAMI components/statistics	Study population (N = 1281)	Urban (n = 649)	Rural (n = 632)	*p* value^*^
**Water/sanitation**				0.889
0, n (%)	6 (0.5)	3 (0.5)	3 (0.5)	
4, n (%)	359 (28.0)	181 (28.6)	178 (27.4)	
8, n (%)	916 (71.5)	448 (70.9)	468 (72.1)	
Overall score, mean (±SD)	7	7, 2	7, 2	0.682**
**Assets** ^**§**^				<0.001
0, n (%)	12 (0.9)	12 (1.9)	0	
1, n (%)	37 (2.9)	37 (5.9)	0	
2, n (%)	60 (4.7)	56 (8.9)	4 (0.6)	
3, n (%)	84 (6.6)	68 (10.8)	16 (2.5)	
4, n (%)	144 (11.2)	102 (16.1)	42 (6.5)	
5, n (%)	205 (16.0)	112 (17.7)	93 (14.3)	
6, n (%)	316 (24.7)	132 (20.9)	184 (28.4)	
7, n (%)	288 (22.5)	66 (10.4)	222 (34.2)	
8, n (%)	135 (10.6)	47 (7.4)	88 (13.6)	
Overall score, mean (SD)	5,2	6, 1	5, 2	<0.001
**Maternal education** ^**‡**^				<0.001
0, n (%)	29 (2.3)	27 (4.3)	2 (0.3)	
3, n (%)	135 (10.5)	111 (17.6)	24 (3.7)	
5, n (%)	195 (15.2)	145 (22.9)	50 (7.7)	
7, n (%)	255 (19.9)	158 (25.0)	97 (14.9)	
8, n (%)	667 (52.1)	191 (30.2)	476 (73.3)	
Overall score, mean (SD)	7, 2	7, 1	6, 2	<0.001
**Income in USD, octiles (range)** ^**‡‡**^				
1 (19.86–105.24), n (%)	10 (0.8)	10 (1.6)	0	<0.001
2 (105.25–166.80), n (%)	19 (1.5)	18 (2.8)	1 (0.2)	
3 (166.81–206.52), n (%)	7 (0.5)	7 (1.1)	0	
4 (206.53–248.22), n (%)	18 (1.4)	18 (2.8)	0	
5 (248.23–327.65), n (%)	43 (3.4)	40 (6.3)	3 (0.5)	
6 (327.66–476.59), n (%)	98 (7.7)	81 (12.8)	17 (2.6)	
7 (476.60–853.89), n (%)	67 (5.2)	60 (9.5)	7 (1.1)	
8 (≥853.90), n (%)	1019 (79.5)	398 (63.0)	621 (95.7)	
Overall score (mean ± SD)^¶^	7 ± 1	8 ± 0	7 ± 2	<0.001
Overall Total WAMI score (mean ± SD)	26.4 ± 4.7	25.7 ± 4.2	18.9 ± 3.9	<0.001
Overall WAMI Index (mean ± SD)	0.8 ± 0.2	0.80 ± 0.1	0.59 ± 0.1	<0.001

n, Frequency; SD, standard deviation; USD, United States dollar; WAMI: water/sanitation, assets, maternal education, and income.

* Chi-square test. **Mann Whitney U test. ^§^ People could have more than one asset of the same category; assets included a refrigerator, bank account, iron, desktop/laptop, radio, sofa, and sewing machine. ^‡^ Number of years of maternal education (0–16 years) divided by 2. ^‡‡^ Based on the August 2024 exchange rate of 1 USD = 25000 VNĐ.

^¶^ Mean octile score.

### HAV seroprevalence

The overall HAV seroprevalence was 69.2%, with 57.9% and 80.7% in urban and rural areas, respectively (p < 0.001) (Table 3).

**Table 3:** HAV seroprevalence between urban and rural areas.

Seroprevalence of total anti-HAV antibodies	Study population (N = 1281)	Urban (n = 649)	Rural (n = 632)	*p v*alue
Reactive, n (%)	886 (69.2)	376 (57.9)	510 (80.7)	<0.001
Non-Reactive, n (%)	395 (30.8)	273 (42.1)	122 (19.3)	

HAV, hepatitis A virus; n, frequency.

**Fig 1:**
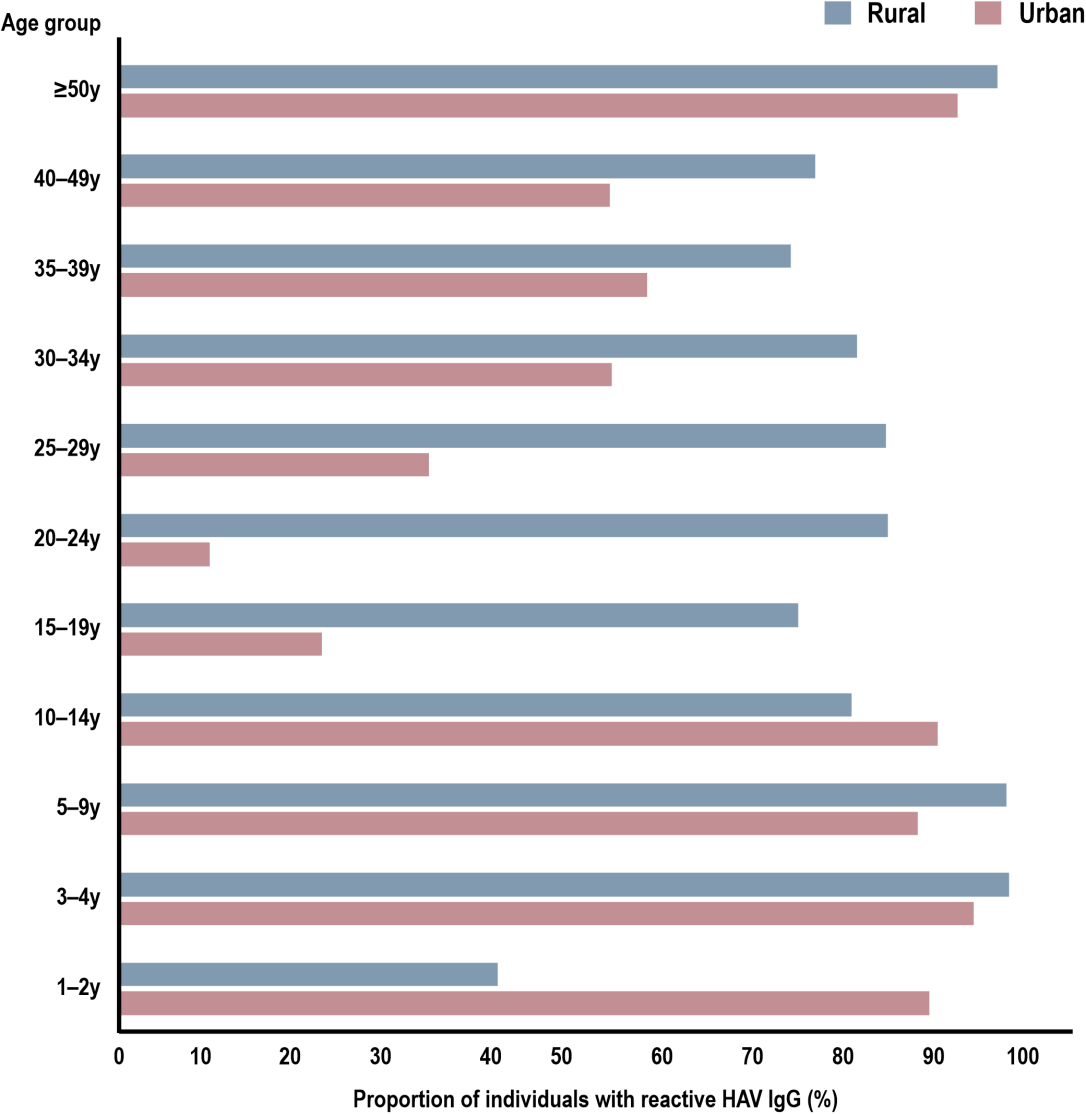
Proportion of Seropositive HAV by age group. HAV, hepatitis A virus.

Seropositivity was relatively constant among age groups in the rural areas compared with urban areas. In the urban sites, there was higher seroprevalence in younger age groups (1–2 and 10–14 years). Among participants aged ≤15 years, particularly those aged ≤2 years, the seropositivity tends to be higher or approximately equal in urban areas compared with rural areas. A considerably higher positivity rate was observed in rural areas than in urban areas for participants starting after 15–19 years (Fig 1).

#### AMPI.

**Fig 2:**
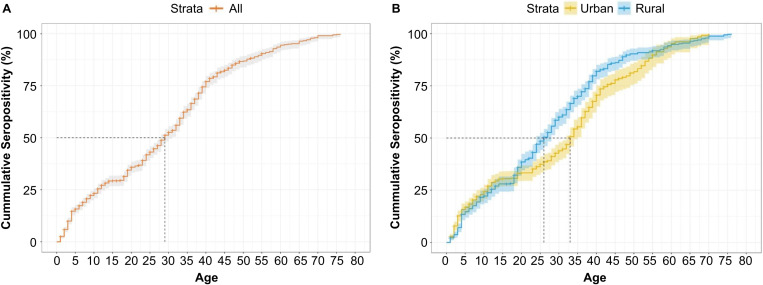
Kaplan-Meier curves for AMPI, Overall (A) and by Region (B). CI, confidence interval; AMPI, age at the midpoint of population immunity.

The estimated AMPI for the overall population was 29 years, indicating a potentially intermediate HAV endemicity in Vietnam. Region-specific AMPIs were 33 years in urban and 26 years in rural areas, (Fig 2).

### Risk factors for HAV seroprevalence

Participants’ responses to each section of the questionnaire were compared to identify differences in potential risk factors for HAV infection between urban and rural areas. Multivariate logistic regression analysis was used to identify statistically significant factors associated with HAV seropositivity for each section.

#### Sociodemographic parameters.

**Fig 3:**
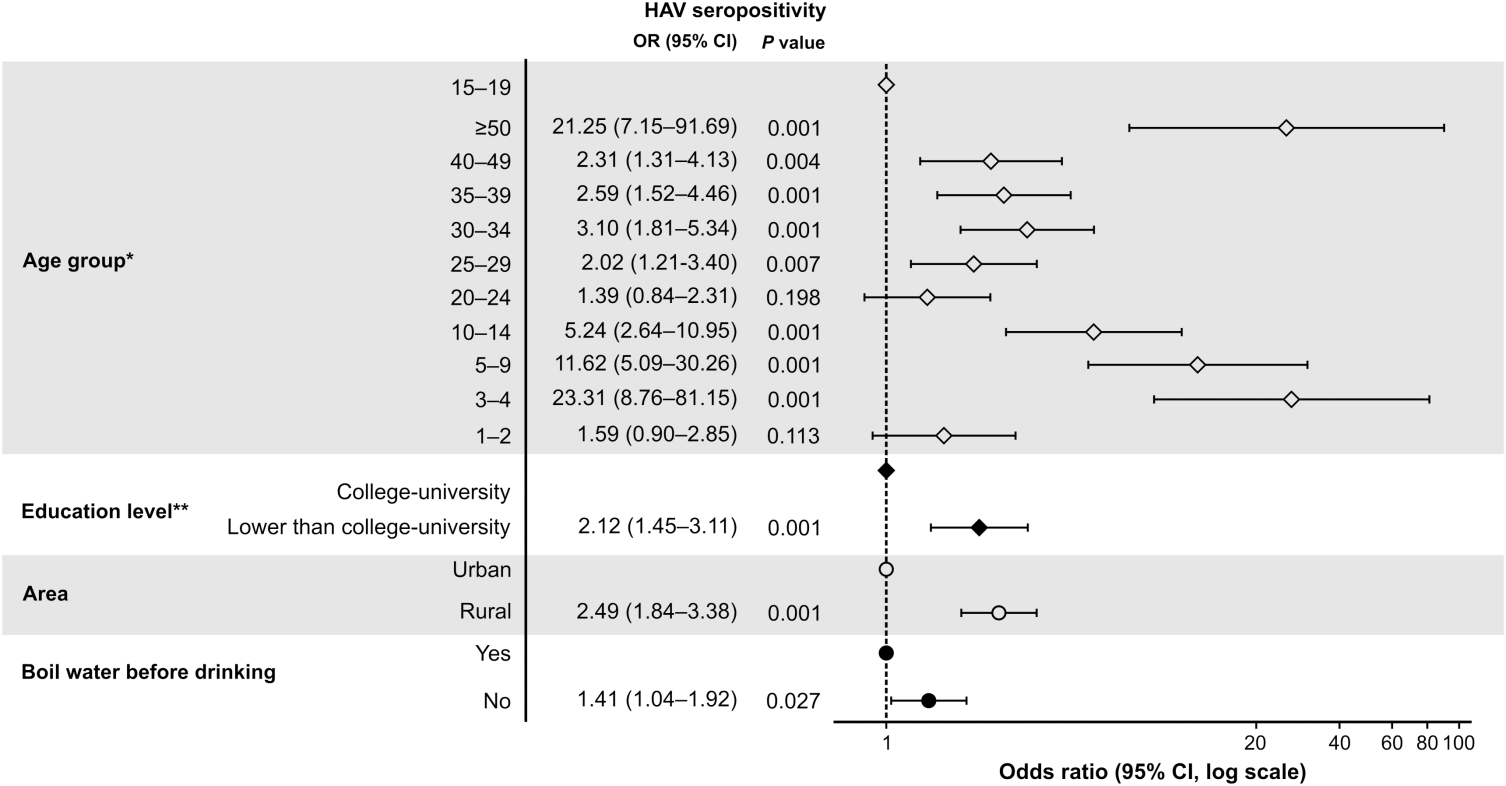
Multivariate regression analysis for factors associated with HAV seropositivity. CI, confidence interval; HAV, hepatitis A virus. *Age group 15-19 years was chosen as the reference group because this group had the lowest rate of HAV seropositivity. **Category “lower than university” adds all the other individual categories (i.e., illiteracy, high school, middle school, primary school, etc.).

Significant differences in sociodemographic parameters between the two geographic regions are summarized in Table 1. Multivariate regression analysis identified age group, education level, living area (rural vs. urban), and the use of boiled drinking water as statistically significant factors associated with HAV seropositivity, (Fig 3). Compared with the 15–19-year age group, seroprevalence was significantly higher in children aged 3–4 years (OR: 23.31, 95% CI 7.8, 69.1, p < 0.001) and adults aged ≥50 years (OR: 21.25, 95% CI 7.1, 91.7, p < 0.001). Lower education levels were associated with higher PRs, with statistical significance observed for participants with less than a college education compared to those with college/university education (OR: 2.12, 95% CI 1.4–3.1, p < 0.001). Participants in urban areas had a significantly lower prevalence of HAV infection than those in rural areas (OR: 2.49, 95% CI 1.84, 3.38, p < 0.001).

#### Hepatitis‑related medical history.

Few respondents reported a medical history of hepatitis, with 4.8% from urban areas and 5.2% from rural areas. A significantly higher proportion of rural respondents had lived in their current area during the first five years of life compared to urban respondents (82.9% vs. 59.8%, p < 0.001). Only 1.6% of participants resided in urban areas during early childhood, while 33.3% were from rural areas (S2 Table). A total of 154 participants had vaccination cards confirming HAV vaccination (109 [16.8%] vs. 45 [7.1%] participants in urban and rural areas, respectively). Among vaccinated participants, 81.8% (126/154) were reactive to anti-HAV total antibodies. Of the 427 child participants (≤15 years), 121 had received hepatitis A vaccine, with significantly higher vaccination coverage in urban areas compared to rural areas (38.4% vs. 18%, p < 0.001); 75/83 (90.4%) in urban areas and 34/38 (89.5%) in rural areas tested positive for anti-HAV total antibodies, (S3 Table).

#### Access to safe water.

There were significant differences in terms of water sources used for drinking and other purposes in urban and rural areas (S4 Table). Overall, 62.4% of urban households used piped water for drinking within their dwellings, compared with 31.6% in rural areas (p < 0.001). Urban participants mainly used piped water (71.5%), while some used bottled water (9.9%), and a few used tubewells (8.7%) for other purposes, such as cooking and handwashing. In contrast, rural areas used different sources (52.9% used piped water, 25.5% tubewells, 6.5% protected dug wells, and 5.2% rainwater). Furthermore, 91.4% of urban participants reported treating their water compared with 86.9% of rural respondents (p = 0.01). Safe drinking water practices were more prevalent in urban areas, with 75.2% of participants boiling water compared to 67.6% in rural regions (p = 0.003). Additionally, 54.4% of urban respondents used a water filter compared with 36.1% of rural respondents (p < 0.001). Composite toilets were exclusively reported in rural areas (26.1%, p < 0.001), whereas 99.4% of urban participants used toilets that flushed into septic tanks, compared with 60.8% in rural areas. Multivariate regression analysis showed that seropositivity was significantly lower among participants who boiled their drinking water (OR: 1.41, 95% CI 1.04, 1.91, p = 0.027) (Fig 3).

#### Hygienic food intake practices.

Significant differences in food preparation and intake were noted between urban and rural areas (S5 Table). A dedicated cooking surface was used in 88.6% of urban households compared with 74.7% in rural households (p < 0.001). Most urban participants (78.4%) typically had meals prepared at home, compared with 56.2% in rural areas (p < 0.001). In both regions, most reported that they ‘always’ or ‘most of the time’ wash their hands before meals and after defecation (p < 0.001). Additionally, 50.5% of urban and 41.6% of rural kitchens were reported to be mostly free from insects and rodents (p = 0.005). However, multivariate analysis showed no significant differences in seropositivity related to food intake practices between urban and rural populations.

#### Knowledge about hepatitis A disease.

A significantly higher proportion of urban participants reported knowledge of HAV compared to their rural counterparts (79.5% vs. 46.7%, p < 0.001) (S6 Table); further, 84.7% of urban respondents identified hepatitis as an infectious disease compared with 68.8% of rural respondents (p < 0.001). Furthermore, 71.1% of urban participants acknowledged that HAV could be transmitted through contaminated food or water compared with 47.8% of the rural participants (p < 0.001). Awareness of the risks associated with consuming contaminated food or water that can lead to HAV infection was also higher among urban respondents than rural respondents (76.0% vs. 47.8%; p < 0.001). Knowledge of vaccine availability against HAV was significantly higher in urban areas (77.5%) compared to rural areas (65.1%; p < 0.001). Additionally, urban participants reported better recognition of HAV symptoms, including jaundice (83.7% vs. 72.2% rural, p < 0.001), abdominal pain (67.8% vs. 54.9, p = 0.001), dark tea-colored urine (63.4% vs. 47.1%, p < 0.001), and fever (65.3% vs. 46.1%, p < 0.001). However, multivariate analysis showed no significant differences in seropositivity based on knowledge of hepatitis A between urban and rural populations. Seroprevalence was significantly lower in participants who were aware that HAV is an infectious disease.

## Discussion

This cross-sectional survey assessed the HAV seroprevalence in urban and rural areas of Vietnam and found significant differences in seropositivity rates and key associated risk factors. The overall HAV seroprevalence of 69.2% observed in this study was lower than the near-complete seroconversion observed in 1994, which classified Vietnam as high endemicity [24]. Recent epidemiological data are limited in Vietnam; however, a survey conducted among immigrant women from Vietnam in Korea (2011–2017) indicated a notable shift in HAV exposure, with seroprevalence of 60% among those aged 20–29 years and 80% among those aged 30–40 years [25]. Findings from the current study indicate a transition from high to intermediate endemicity, with 50% of the population showing prior HAV infection at an older age of 29 years. Seroprevalence was notably higher in rural areas (80.7%) compared to urban areas (57.9%), consistent with global trends of higher HAV burden in rural populations, likely due to lower socioeconomic status and inadequate sanitation [6,7,26]. These findings suggest a changing landscape of HAV epidemiology in Vietnam, aligning with trends observed in other Southeast Asian countries [10–12].

Several low- and lower-middle-income countries worldwide have experienced a shift in hepatitis A epidemiology over the past decade. Southeast Asian countries have also transitioned from higher to lower endemicity [8,10,27]. In the Philippines, the AMPI increased from 20 years in 1996–36 years in 2023, indicating a move toward low endemicity [12]. Similarly, in Thailand, the AMPI increased from 4.5 years in 1971 to 42.0 years in 2014 [9]. Further, studies from Laos and Indonesia [13] and Laos [28] also indicate a similar upward trend for seroconversion.

The findings of this study highlight significant trends in HAV exposure among different age groups and geographic areas in Vietnam. Improvements in hygiene and sanitation in urban areas of Vietnam may explain the lower seroprevalence observed among younger age groups (1–2 and 10–14 years) and the difference in AMPI between urban and rural areas (33 years vs. 26 years). We also observed distinct patterns in the proportion of HAV-positive participants across different age groups, with seroprevalence <60% in young adults (15–29 years) and 85% in children (<10 years). Higher seroprevalence among urban young adults is likely due to advancements in public health awareness and vaccination coverage. Additionally, ongoing rural-to-urban migration may influence age-specific seroprevalence, as individuals residing in rural areas for the past five years showed significantly higher seroprevalence, reflecting persistent endemic conditions in those regions. Furthermore, rapid urbanization and economic growth in Vietnam pose challenges in understanding the dynamics of HAV transmission. The difference in AMPI between urban and rural areas further reflects geographic and sociodemographic differences in HAV epidemiology. These findings suggest an epidemiological transition, with reduced childhood exposure to HAV among urban populations and a high disease burden among rural populations.

The Vietnamese government recommends Hepatitis A vaccination for children aged 1–5 years [14]. However, access to vaccines in rural areas may be limited, as they are primarily available in private healthcare settings. We observed HAV vaccination coverage was significantly higher among children (≤15 years) in urban compared to rural areas. Seroprevalence among vaccinated children was high in urban areas (90.4%), particularly those aged <2 years, likely reflecting improved vaccine access and uptake. In contrast, a high seroprevalence was observed in adults >20 years in rural areas, likely due to past infections from greater exposure and lower vaccination rates. This may explain the persistent burden of HAV transmission in these rural areas. Notably, these rates are higher than those reported in a previous study in Vietnam [24], which found IgG anti-HAV prevalence to be 39.2% and 10.5% in children from rural and urban areas, respectively. Various sociodemographic factors and behaviors related to hygiene, food preparation, and water sanitation were associated with HAV seroprevalence, further indicating the shift in HAV epidemiology in Vietnam.

Hepatitis A endemicity correlated with socioeconomic factors, with higher endemicity reported in middle-income countries and lower endemicity in high-income countries [5,7]. Socioeconomic status is often associated with access to clean drinking water, sanitation, education, and income levels [6,10,13,29,30]. In this study, significant factors associated with high seroprevalence included age group, education level, residing areas (urban or rural), and practice of boiling water for drinking.

Educational level and disease knowledge often reflect socioeconomic status, influencing access to clean water, sanitation, and awareness of disease transmission. Seroprevalence was expectedly low among participants with college or university education in this study, consistent with previous studies [10,31,32]. However, seroprevalence was high among participants who were aware that HAV is an infectious, vaccine-preventable disease. This suggests that knowledge alone is insufficient for prevention, and other factors, including accessible resources, such as vaccines, clean water, and proper sanitation facilities are essential to support disease awareness. Public health strategies should, therefore, integrate education with practical, tangible measures to prevent disease transmission effectively.

In this study, seroprevalence was high among participants who lived in rural areas during their first five years, suggesting that early-life socioeconomic conditions may increase the risk of HAV infection later in life. Addressing these early-life disparities could improve health outcomes for populations with rural backgrounds. While the WAMI index indicated no significant differences in water and sanitation access between urban and rural areas, urban participants were more likely to use boiling as a water purification method. Further, multivariate analysis found a significant association between not boiling water for drinking and high seroprevalence in rural areas. These findings suggest that, despite similar access to water and sanitation, urban and rural communities differ notably in their water safety practices. Notably, the Multiple Indicator Cluster Survey conducted in 2014 in Vietnam reported that only 6% of households in the poorest quintile had access to piped water compared to 68% in the richest, which may increase the HAV burden among poor dwellings [33]. Therefore, promoting safe water practices in rural areas may help improve public health and mitigate disparities in HAV infection rates.

Hepatitis A infection is a serious health concern owing to the severity of clinical outcomes and high costs associated with the management of the disease. The clinical outcomes of HAV infection include relapsing hepatitis, prolonged cholestasis, sepsis/septic shock, hepatic encephalopathy, acute liver failure and death [34–36]. Evidence from the literature illustrates an increase in the mean age of infection, with implications of more severe and serious outcomes in older age groups [37]. Even though the management of infection is limited to supportive care and symptomatic treatment, HAV infection is a vaccine-preventable disease. Although effective vaccines are available worldwide, immunization strategies differ [38]. The WHO recommends universal childhood vaccination in intermediate to low-endemicity areas if there is a rise in acute cases, a shift in endemicity, or favorable cost-effectiveness analyses [39]. Findings from this study indicate a shift in endemicity, with varying HAV seroprevalence rates by geographic and socioeconomic factors, highlighting the importance of targeted vaccination strategies. However, we did not assess clinical outcomes, acute case incidence, or healthcare resource use related to hepatitis A. Evaluating these factors could provide valuable insights for cost-effectiveness models, as seen in Indonesia [40], which found that universal childhood hepatitis A immunization is a cost-effective intervention. Future research should examine HAV-related healthcare utilization and disease burden to support cost-effectiveness models for hepatitis A vaccination in Vietnam.

Our study has some inherent limitations of observational design and nonrandom sampling. Findings were based on population from only two geographic areas, and the age-stratified approach may limit generalizability to the broader Vietnamese population, where variations in demographics and socioeconomic factors could influence HAV prevalence. Furthermore, response bias may have affected the reliability of the data, as participants could have provided socially desirable answers to sensitive questions regarding income, eating habits, and hygienic behaviors. Documentation of received vaccines was incomplete, and vaccination status might be underreported.

## Conclusions

Vietnam has potentially experienced a shift towards intermediate HAV endemicity. This epidemiological shift may result in increased susceptibility to Hepatitis A infections among adolescents and adults, who are at higher risk of experiencing severe disease outcomes later in life. Key risk factors for HAV infection include age group, educational level, residing area, and the practice of boiling water to ensure safe drinking. These factors highlight the crucial role of public health interventions and targeted vaccination strategies in addressing hepatitis A infection, particularly among high-risk groups.

## Supporting information

S1 Appendix Survey Questionnaire. (DOCX)

S1 Table Theoretical sample size by age group. (DOCX)

S2 Table Comparison of past medical history factors between urban and rural areas. (DOCX)

S3 Table Vaccination status seroprevalence in participants ≤ 15 years of age. (DOCX)

S4 Table Comparison of water safety access factors between urban and rural areas. (DOCX)

S5 Table Comparison of hygienic food intake factors between urban and rural areas. (DOCX)

S6 Table Comparison of knowledge factors on HAV disease between urban and rural areas. (DOCX)
