# Supplementary material for: Seroprevalence of hepatitis A virus infection in urban and rural areas in Vietnam
Source: PLoS One. 2025 May 16;20(5):e0323139. doi: 10.1371/journal.pone.0323139 (PMC12084049; doi:10.1371/journal.pone.0323139)
Supplement: S1 Appendix — (DOCX) [file pone.0323139.s001.docx]

[**S1 Appendix.**](https://journals.plos.org/plosone/article/file?type=supplementary&id=10.1371/journal.pone.0315831.s001)**Survey Questionnaire.**

**Section A. Sociodemographic**

**Please use a circle to mark the answer given by the participant/parent**

| **A1** | Education of 12+ participant:   1. Primary School (6years) 2. Middle School (9 years) 3. High School (13 years) 4. Graduate/post-graduate (16+ years) 5. Illiterate 6. <6y (for participants <12y age) |
| --- | --- |
| **A2** | Occupation of participant:  1. Professional 2. Semi-professional 3. Clerical/shop owner 4. Skilled worker 5. Semi-skilled worker 6. Unskilled worker  7. Household duties 8. Unemployed (adults) 9.Attending School  10. Attending Garderie/Pre-School 11. At home (children) |
| **A3** | For all participants, What was the highest education attained from you parents of parents choose the option number as mentioned in A1  (a) Education of Father: 1- 2- 3- 4- 5- 6-  (b) Education of Mother: 1- 2- 3- 4- 5- 6- |
| **A4** | Total number of family members living in the same household: _________ |
| **A5** | *Total family monthly income _________________*  *(local currency, please consider to include multiple selection accordingly to local scenario:* |
| **A6** | Regarding your households walls roof and floor are made of (please choose one)   - Permanent building materials are used for walls, roof and floor - Natural/Traditional/ Rudimentary materials |
| **A7** | Do you have at home (you can tick more than one option   \| - Fridge/Refrigerator - Bank account - Iron - Desktop/laptop - Radio - Sofa - Sewing machine \| \| --- \| |
| **A8** | How many habitable rooms your household has? ______________ |
| **Section B . Knowledge of disease**  *(Participants 14+* ***should answer by themselves*** *these Section of question in not otherwise specified)* | |
| **B1** | Have you heard about a disease called Hepatitis A before? 1. Yes 2. No  *(If the answer is “No” then go to the Section C)* |
| **B2** | \| What kind of a disease is it? \| 1.Communicable \| \| --- \| --- \| \| 2.Non-Communicable \| \| 3. Do not know \| |
| **B3** | \| What is the main way of transmission? \| 1. By blood \| \| --- \| --- \| \| 1. By air \| \| 1. Sexually Transmitted \| \| 1. By contaminated food/water \| \| 1. By mosquito bite \| |
| **B4** | \| Is there a vaccine to prevent Hepatitis A  available in __(Name of country)______? \| 1. Yes \| \| --- \| --- \| \| 1. No \| \| 1. Do not know \| |
| **B5**  **B6** | \| \| What are the possible risk factor/ factors?  (Multiple answers are allowed) \| \| - Use unclean toilets \| \| \|  \| \| --- \| --- \| --- \| --- \| --- \| --- \| \| - Consume contaminated water/ food \| \| \|  \| \| - Talking to ill \| \| \|  \| \| - Sharing a room with an infected individual \| \| \|  \| \| What are the possible symptoms of Hepatitis A? \| \| \| \| \| a. Yellowish discoloration of eyes \| Yes \| \| No \| Don’t know \| \| \| b. Abdominal pain \| Yes \| \| No \| Don’t know \| \| \| c. Nasal bleeding \| Yes \| \| No \| Don’t know \| \| \| d. Dark tea colour urine \| Yes \| \| No \| Don’t know \| \| \| e. Numbness over extremities \| Yes \| \| No \| Don’t know \| \| \| f. Fever \| Yes \| \| No \| Don’t know \| \| \| g. Pale stools \| Yes \| \| No \| Don’t know \| \| \| \| --- \| --- \| --- \| --- \| --- \| --- \| --- \| --- \| --- \| --- \| --- \| --- \| --- \| --- \| --- \| --- \| --- \| --- \| --- \| --- \| --- \| --- \| --- \| --- \| --- \| --- \| --- \| --- \| --- \| --- \| --- \| --- \| --- \| --- \| --- \| --- \| --- \| --- \| --- \| --- \| --- \| --- \| --- \| --- \| --- \| --- \| --- \| --- \| --- \| --- \| --- \| --- \| --- \| --- \| --- \| --- \| --- \| --- \| --- \| --- \| --- \| --- \| --- \| --- \| --- \| |
|  |  |
| **Section C. Past Medical History of Hepatitis**  *(participants 14+ can answer this section questions with the assistance of their guardian)* | |
| **C1**  **C1a**  **C1b**  **C1c**  **C1d** | Have you (your child) ever been diagnosed with Hepatitis Disease? 1. Yes 2. No 3. DK  (a ) If Yes, what type? ____________, if No go to Question C2  (*Write the answer provided, if the answer is Don’t Know, write down DK)*  (b) if Yes, were you living in the same household as today? 1. Yes 2. No  (c) if No to the last question, where were you living? (Village Name) ________________  (d) What was the Area of Residence of your household  when got infected? 1. Urban 2. Rural |
| **C2a**    **C2b** | (a) Did you (your child) live the most during your first 5 years of life in  the current village? 1. Yes 2. No  (b) What was the Area of Residence of your household at that time: 1. Urban 2. Rural |
| **C3a**  **C3b** | Has anyone in your family got jaundice with fever in the past? 1. Yes 2. No   1. If yes, who? ______________ (mother /father/siblings /others (specify)) 2. When (at least the year of occurrence. If more than one member affected record the earliest reported date)? MM/YEAR |
| **C4a**  **C4b** | Have you ever been vaccinated against Hepatitis A? 1. Yes 2. No  *Confirm the answer by checking vaccination card?? 1. Yes 2. No* |
| **Section D . Drinking Water Safety**  **(***participants 14+ can answer this section questions with the assistance of their guardian)* | |
| **D1** | What is the main source of drinking-water for members of your household?  *(If answer is #10 go to D2, otherwise jump to D3)* |
|  | \| 1. Piped water into dwelling 2. Piped water to yard/plot 3. Public tap/ standpipe 4. Tubewell/   bore-hole   1. Protected dug well \| 1. Unprotected dug well 2. Protected spring 3. Unprotected spring 4. Rainwater collection 5. Bottled water \| 1. Cart with small tank/drum 2. Tanker-truck 3. Surface water (river, dam, lake, pond, stream, canal, irrigation channels) 4. Other (specify)   _____________ \| \| --- \| --- \| --- \| |
| **D2** | You indicated that bottled water was your main source of drinking water, now I will like to ask you what is the main source of water used by your household **for other purposes, such as cooking and hand washing?**   \| 1. Piped water into dwelling 2. Piped water to yard/plot 3. Public tap/ standpipe 4. Tubewell/   bore-hole   1. Protected dug well \| 1. Unprotected dug well 2. Protected spring 3. Unprotected spring 4. Rainwater collection 5. Bottled water \| 1. Cart with small tank/drum 2. Tanker-truck 3. Surface water (river, dam, lake, pond, stream, canal, irrigation channels) 4. Other (specify)   _____________ \| \| --- \| --- \| --- \| |
| **D3** | How long does it take to go there, get water, and come back?  #. of minutes _______________ ,  *(Ask for his/her best guess, otherwise please write down DK if participant don’t know the answer to this question or 0 (zero) if water source is within the premises of the household)* |
| **D4** | Do you treat your water in any way to make it safer to drink? 1. Yes 2. No |
| **D5** | What do you usually do to the water to make it safer to drink?  Record all items mentioned   - Boil - Add bleach/chlorine - Strain it through a cloth - Use a water filter (ceramic, sand, composite, etc.) - Solar disinfection - Let it stand and settle - Other (specify) - Don’t Know |
| **D6** | What kind of toilet facility do members of your household usually use?  If “flush” or “pour flush” probe: Where does it Flush/pour flush to: |

| \|  \| 1. Piped sewer system 2. septic tank 3. pit latrine 4. elsewhere 5. unknown place/not sure/ 6. Ventilated improved pit latrine (VIP) \| 1. Pit latrine with slab 2. Pit latrine without slab/open pit 3. Composting toilet 4. Hanging toilet/hanging latrine 5. No facilities or bush or field 6. Other (specify) \| \| \| --- \| --- \| --- \| --- \| \| **D7** \| How many households use this toilet facility? __________  *(please write down 0 (cero) if the toilet is not shared with any other household or DK if the participant don’t know an answer for this question* \| \| \| \| **D8** \| Can any member of the public use this toilet? 1. Yes 2. No 3. DK \| \| \| \| **D9** \| *This question applies to participants <4y age.*  *Th*e last time [name of youngest child] passed stools, what was done to dispose of the stools*?* \| \| \| \| 1. Child used toilet/latrine 2. Put/rinsed into toilet or latrine 3. Put/rinsed into drain or ditch 4. Thrown into garbage \| \| 1. Buried 2. Left in the open 3. Other (specify)__________ 4. DK \|   **Section E. HYGIENIC FOOD INTAKE**  *participants 14+ can answer this section questions with the assistance of their guardian* | | | | | |
| --- | --- | --- | --- | --- | --- | --- | --- | --- | --- | --- | --- | --- | --- | --- | --- | --- | --- | --- | --- | --- | --- | --- | --- | --- |
| **E1** | Where do you prepare your food at home? 1. On the ground  2. multi-purpose table  3. Table exclusively set for cooking | | | | |
| *Please answer the following questions based on what you have done during the past 7 days* | | | | | |
|  |  | **Category** | | | |
|  | **Question:** | **Never** | **Sometimes** | **Most of the time** | **Always** |
| **E2** | Did you get your main meals from home? |  |  |  |  |
| **E3** | Did you get your main meals from street? |  |  |  |  |
| **E4** | Did you wash your hands before handling food? |  |  |  |  |
| **E5** | Did you wash your hands before eating food? |  |  |  |  |
| **E6** | Did you wash your hands after defecation?  *(practice of mother should be asked in case of younger children)* |  |  |  |  |
| **E7** | Was the kitchen which prepared the food you ate, free of insects and rodents? |  |  |  |  |

You have completed your study participation. Please acknowledge the time and interest to participate into the study and provide information leaflet of Hepatitis A disease, please follow the stablished procedure to indicate the participant how to get the compensation agreed and the study result if its demanded by the Ethics committee.
