# Supplementary material for: Seroprevalence of hepatitis A virus infection in urban and rural areas in Vietnam
Source: PLoS One. 2025 May 16;20(5):e0323139. doi: 10.1371/journal.pone.0323139 (PMC12084049; doi:10.1371/journal.pone.0323139)
Supplement: S1 Table — (DOCX) [file pone.0323139.s002.docx]

**S1 Table.** **Theoretical sample size by age group**

| **Age Group** | **Expected HAV Seroprevalence Prevalence** | **Sample size needed n=** | **Safe Margin of error n=** |
| --- | --- | --- | --- |
| 1-2y | 0.1 | 50 | 55 |
| 3-4y | 0.1 | 50 | 55 |
| 5-10y | 0.2 | 44 | 50 |
| 10-14y | 0.2 | 44 | 50 |
| 15-19y | 0.4 | 65 | 70 |
| 20-24 | 0.5 | 68 | 75 |
| 25-29 | 0.5 | 68 | 75 |
| 30-34 | 0.7 | 57 | 65 |
| 35-39 | 0.7 | 57 | 65 |
| 40-49 | 0.8 | 44 | 50 |
| 50+ | 0.9 | 25 | 30 |
| **Total** |  | **572** | **640** |
