# Supplementary material for: Seroprevalence of hepatitis A virus infection in urban and rural areas in Vietnam
Source: PLoS One. 2025 May 16;20(5):e0323139. doi: 10.1371/journal.pone.0323139 (PMC12084049; doi:10.1371/journal.pone.0323139)
Supplement: S2 Table — (DOCX) [file pone.0323139.s003.docx]

**S2 Table.** **Comparison of past medical history factors between urban and rural areas**

| **Past medical history of hepatitis** | **Urban n (%)** | **Rural n (%)** | **p-value** |
| --- | --- | --- | --- |
| **Have you (your child) ever been diagnosed with Hepatitis Diseases** | | | |
| yes | 31 (4.8) | 33 (5.2) | <0.001 |
| Hepatitis C | 4 | 3 |  |
| Hepatitis B | 20 | 27 |  |
| No | 565 (87.1) | 470 (74.4) |  |
| Unknown | 53 (8.2) | 129 (20.4) |  |
| **Living in the same household as today** | | | |
| Yes | 20 | 26 | NA |
| No | 4 | 4 |  |
| **Lived the most during first 5 years of life in current area** | | | |
| yes | 388 (59.8) | 524 (82.9) |  |
| no | 261 (40.2) | 108 (17.1) | <0.001 |
| **Area of residence at that time** | | | |
| Urban | 10 (1.6) | 427 (33.3) | <0.001 |
| Rural | 232 (35.7) | 622 (98.4) |  |
| **Vaccinated to HAV (**vaccination card confirmation) |  |  |  |
| Yes | 109 (16.8) | 45 (7.1) | <0.001 |
| No | 540 (83.2) | 587 (92.9) |  |

NA: not Applicable
