# Supplementary material for: Seroprevalence of hepatitis A virus infection in urban and rural areas in Vietnam
Source: PLoS One. 2025 May 16;20(5):e0323139. doi: 10.1371/journal.pone.0323139 (PMC12084049; doi:10.1371/journal.pone.0323139)
Supplement: S3 Table — (DOCX) [file pone.0323139.s004.docx]

**Table S3. Vaccination status seroprevalence in participants ≤ 15 years of age**

|  | **Urban n (%)** | **Rural n (%)** | **Total**  **n (%)** |
| --- | --- | --- | --- |
| **HAV vaccination** |  |  |  |
| Yes | 83 (38.4) | 38 (18.0) | 121 (28.3) |
| No | 133 (61.6) | 173 (82.0) | 306 (71.7) |
| Total | 216 | 211 | 427 |
| **Positive Anti HAV total by age group (≤ 15 years of age)** | | | |
| 1-2y | 19 (86.4) | 1 (50.0) | 20 |
| 3-4y  5-9y  10-14y  15-19y*  Total** | 25 (96.2)  16 (88.9)  14 (93.3)  1 (50.0)  75 (90.4) | 12 (92.3)  9 (100)  12 (85.7)  0 (0.0)  34 (89.5) | 37  25  26  1  109 |

**^*^**2 subjects were 15 years of age (Urban); **The percentages are calculated based on the proportion of negative results relative to the total count across all age groups. HAV: hepatitis A virus; Yoa: Year of age.
