# Supplementary material for: Seroprevalence of hepatitis A virus infection in urban and rural areas in Vietnam
Source: PLoS One. 2025 May 16;20(5):e0323139. doi: 10.1371/journal.pone.0323139 (PMC12084049; doi:10.1371/journal.pone.0323139)
Supplement: S4 Table — (DOCX) [file pone.0323139.s005.docx]

**S4 Table.** **Comparison of water safety access factors between urban and rural areas**

| **Features of water safety access** | **Urban (n=649)** | **Rural (n=632)** | **p- value** |
| --- | --- | --- | --- |
| **Main source of drinking water for household members** | | | |
| piped water in the dwelling | 405 (62.4) | 200 (31.6) |  |
| pipe water to yard/plot | 2 (0.3) | 6 (0.9) |  |
| public tap/standpipe | 0 | 1 (0.2) |  |
| tubewell | 27 (4.2) | 117 (18.5) |  |
| protected dug well | 14 (2.2) | 55 (8.7) |  |
| unprotected dug well | 1 (0.2) | 6 (0.9) |  |
| protected spring | 6 (0.9) | 6 (0.9) |  |
| unprotected spring | 1 (0.2) | 1 (0.2) |  |
| bottled water | 172 (26.5) | 153 (24.2) | <0.001 |
| rainwater collection | 16 (2.5) | 76 (12) |  |
| cart with small tank | 5 (0.8) | 6 (0.9) |  |
| other, unspecified | 0 | 5 (0.8) |  |
| **Supplies of water for other household purposes (cooking, handwashing)** | | | |
| piped water into dwelling | 123 (71.5) | 81 (52.9) |  |
| pipe water into yard | 1 (0.6) | 0 |  |
| tubewell | 15 (8.7) | 39 (25.5) |  |
| protected dug well | 9 (5.2) | 10 (6.5) |  |
| unprotected dug well | 1 (0.6) | 1 (0.7) |  |
| protected spring | 2 (1.2) | 4 (2.6) |  |
| bottled water | 17 (9.9) | 4 (2.6) | <0.001 |
| cart with small tank | 1 (0.6) | 2 (1.3) |  |
| rainwater collection | 3 (1.7) | 8 (5.2) |  |
| other | 0 | 4 (2.6) |  |
| **Treat water** | | | |
| Yes | 593 (91.4) | 549 (86.9) |  |
| No | 56 (8.6) | 83 (13.1) | 0.01 |
| **Safe drinking water practices** | | | |
| Boil | 488 (75.2) | 427 (67.6) | 0.003 |
| Add bleach /chlorine | 19 (2.9) | 16 (2.5) | 0.664 |
| Use a water filter | 353 (54.4) | 228 (36.1) | <0.001 |
| Solar disinfection | 39 (6.0) | 28 (4.4) | 0.204 |
| Let it stand and still | 53 (8.2) | 78 (12.3) | 0.014 |
| **Type of toilet facility** | | | |
| composite toilet | 0 | 165 (26.1) | <0.001 |
| hanging toilet | 0 | 7 (1.1) |  |
| **Where does it flush/pour flush?** | | | |
| piped sewer system | 3 (0.5) | 9 (1.4) |  |
| septic tank | 645 (99.4) | 384 (60.8) |  |
| elsewhere | 0 | 2 (0.3) |  |
| unknown place | 0 | 2 (0.3) |  |
| ventilated improved pit latrine | 1 (0.2) | 8 (1.3) | NA |
| pit latrine | 0 | 40 (6.3) |  |
| pit latrine with slab | 0 | 10 (1.6) |  |
| pit latrine without slab | 0 | 5 (0.8) |  |

NA: not Applicable
