# Supplementary material for: Seroprevalence of hepatitis A virus infection in urban and rural areas in Vietnam
Source: PLoS One. 2025 May 16;20(5):e0323139. doi: 10.1371/journal.pone.0323139 (PMC12084049; doi:10.1371/journal.pone.0323139)
Supplement: S5 Table — (DOCX) [file pone.0323139.s006.docx]

**S5 Table. Comparison of hygienic food intake factors between urban and rural areas**

| **Hygiene food intake practices** | **Urban (n=649)** | **Rural (n=632)** | **p- value** |
| --- | --- | --- | --- |
| **Where food is prepared at home** | | | |
| On the ground | 7 (1.1) | 77 (12.2) | <0.001 |
| Multi-purpose table | 67 (10.3) | 83 (13.1) |  |
| Table exclusively set for cooking | 575 (88.6) | 472 (74.7) |  |
| **Get main meals from home** | | | |
| Never | 13 (2.0) | 3 (0.5) | <0.001 |
| Sometimes | 87 (13.4) | 49 (7.8) |  |
| Most of the time | 509 (78.4) | 355 (56.2) |  |
| Always | 40 (6.2) | 225 (35.6) |  |
| **Get main meal bought outside** | | | |
| Never | 40 (6.2) | 225 (35.6) | <0.001 |
| Sometimes | 509 (78.4) | 355 (56.2) |  |
| Most of the time | 87 (13.4) | 49 (7.8) |  |
| Always | 13 (2.0) | 3 (0.5) |  |
| **Wash hands before preparing food** | | | |
| Never | 3 (0.5) | 6 (0.9) | <0.001 |
| Sometimes | 34 (5.2) | 54 (8.5) |  |
| Most of the time | 171 (26.3) | 209 (33.1) |  |
| Always | 441 (68.0) | 363 (57.4) |  |
| **Wash hands before eating food** | | | |
| Never | 4 (0.6) | 17 (2.7) | <0.001 |
| Sometimes | 46 (7.1) | 63 (10.0) |  |
| Most of the time | 185 (28.5) | 215 (34.0) |  |
| Always | 414 (63.8) | 337 (53.3) |  |
| **Wash hands after defecation** | | | |
| Never | 1 (0.2) | 8 (1.3) | <0.001 |
| Sometimes | 8 (1.2) | 17 (2.7) |  |
| Most of the time | 97 (14.9) | 181 (28.6) |  |
| Always | 543 (**83.7**) | 426 (67.4) |  |
| **Kitchen where food was prepared free of insects and rodents** | | | |
| Never | 60 (9.2) | 56 (8.9) | 0.005 |
| Sometimes | 132 (20.3) | 173 (27.4) |  |
| Most of the time | 328 (50.5) | 263 (41.6) |  |
| Always | 129 (19.9) | 140 (22.2) |  |
