# Supplementary material for: Seroprevalence of hepatitis A virus infection in urban and rural areas in Vietnam
Source: PLoS One. 2025 May 16;20(5):e0323139. doi: 10.1371/journal.pone.0323139 (PMC12084049; doi:10.1371/journal.pone.0323139)
Supplement: S6 Table — (DOCX) [file pone.0323139.s007.docx]

**S6 Table. Comparison of knowledge factors on HAV disease between urban and rural areas**

| **Attributes of knowledge** | **Urban N (%)** | **Rural N (%)** | **p-value** |  |
| --- | --- | --- | --- | --- |
|  |  |  |  |  |
| **Have heard about HAV** | | | |  |
| Yes | 516 (79.5) | 295 (46.7) | <0.001 |  |
| No | 133 (20.5) | 470 (36.7) |  |  |
| **Kind of disease (n=811)** | | | |  |
| Communicable | 437 (84.7) | 203 (68.8) | <0.001 |  |
| Non-Communicable | 38 (7.4) | 38 (12.9) |  |  |
| unknown | 41 (7.9) | 54 (18.3) |  |  |
| **Main way of transmission** | | | |  |
| By blood | 128 (24.8) | 122 (41.4) | <0.001 |  |
| By air | 4 (0.8) | 9 (3.1) |  |  |
| Sexually Transmitted | 10 (1.9) | 5 (1.7) |  |  |
| By contaminated food/water | 367 (71.1) | 141 (47.8) |  |  |
| By mosquito bite | 2 (0.4) | 3 (1.0) |  |  |
| unknown | 5 (1.0) | 15 (5.1) |  |  |
| **Is there a vaccine to prevent Hepatitis A available in Vietnam?** | | | |  |
| Yes | 400 (77.5) | 192 (65.1) | <0.001 |  |
| No | 56 (10.9) | 36 (12.2) |  |  |
| Unknown | 60 (11.6) | 67 (22.7) |  |  |
| **Possible risk factor/s** | | | |  |
| Use unclean toilets | 213 (41.3) | 104 (35.3) | 0.23 |  |
| Consume contaminated water/ food | 392 (76.0) | 172 (58.3) | <0.001 |  |
| Talking to ill participants | 66 (12.8) | 37 (12.5) | 0.905 |  |
| Sharing a room with infectant individual | 196 (38.0) | 96 (32.5) | 0.259 |  |
| **Possible symptoms of Hepatitis A (yes)** | | | |  |
| Yellowish discoloration of eyes | 432 (83.7) | 213 (72.2) | <0.001 |  |
| Abdominal pain | 350 (67.8) | 162 (54.9) | 0.001 |  |
| Nasal bleeding | 57 (11.0) | 22 (7.5) | 0.212 |  |
| Dark tea colour urine | 327 (63.4) | 139 (47.1) | <0.001 |  |
| Extremities numbness | 63 (12.2) | 35 (11.9) | 0.024 |  |
| Fever | 337 (65.3) | 136 (46.1) | <0.001 |  |
| Pale stools | 196 (38.0) | 105 (35.6) | 0.006 |  |
